# Separating the effects of childhood and adult body size on inflammatory arthritis: a Mendelian randomisation study

**DOI:** 10.1136/rmdopen-2022-002321

**Published:** 2022-08-22

**Authors:** Sizheng Steven Zhao, John Bowes, Anne Barton, George Davey Smith, Tom Richardson

**Affiliations:** 1 Centre for Epidemiology Versus Arthritis, Division of Musculoskeletal and Dermatological Sciences, School of Biological Sciences, Faculty of Biology Medicine and Health, The University of Manchester, Manchester Academic Health Science Centre, Manchester, UK; 2 Medical Research Council Integrative Epidemiology Unit at the University of Bristol, Bristol, UK; 3 Centre for Genetics and Genomics Versus Arthritis, Division of Musculoskeletal and Dermatological Sciences, School of Biological Sciences, Faculty of Biology Medicine and Health, The University of Manchester, Manchester Academic Health Science Centre, Manchester, UK; 4 Population Health Sciences, Bristol Medical School, University of Bristol, Bristol, UK; 5 Novo Nordisk Research Centre, Headington, Oxford, UK

**Keywords:** Childhood obesity, body mass index, inflammatory arthritis, psoriatic arthritis, systemic lupus erythematosus

## Abstract

**Objectives:**

Using Mendelian randomisation (MR), we examined whether childhood body size affects risk of rheumatoid arthritis (RA), ankylosing spondylitis (AS), psoriatic arthritis (PsA), gout and systemic lupus erythematosus (SLE) after accounting for the effect of adult body size.

**Methods:**

Genetic instruments for childhood (age 10 years) and adult body size were derived using data from 453 169 individuals from the UK Biobank study (313 and 580 variants respectively), which have been previously validated using body mass index data from three independent populations. Genome-wide association data comprised 22 350 RA, 9069 AS, 3609 PsA, 13 179 gout and 5201 SLE cases. For each outcome, we conducted univariable MR to estimate the total effects of childhood and adult body size, and multivariable MR to examine the independent effect of childhood body size after accounting for adult body size.

**Results:**

Genetically predicted childhood body size had a total effect on risk of PsA (OR 2.18 per change in body size category; 95% CI 1.43 to 3.31), gout (OR 2.18; 95% CI 1.43 to 3.31) and SLE (OR 2.44; 95% CI 1.14 to 5.22), but not RA (OR 0.95; 95% CI 0.70 to 1.29) or AS (OR 0.96; 95% CI 0.61 to 1.52). After accounting for adult body size, the direct effect of childhood body size was little changed for PsA (OR 1.92; 1.14 to 3.25) and SLE (OR 2.69; 1.24 to 5.87) but was attenuated for gout (OR 1.40; 95% CI 0.94 to 2.09).

**Conclusions:**

Our findings suggest that, for PsA and SLE, the risk conferred from having a larger body size during childhood may not be fully reversable even when a healthy size is achieved in adulthood.

**WHAT IS ALREADY KNOWN ON THIS TOPIC**

- Obesity is strongly associated with inflammatory arthritis, but most studies to date have only examined body size in adulthood. Whether childhood obesity has a direct and long-term influence on risk of inflammatory arthritis is under studied.

**WHAT THIS STUDY ADDS**

- Using a genetic instrumental variable approach, we found that having a larger body size in childhood increases risk of psoriatic arthritis (PsA), gout and systemic lupus erythematosus (SLE), but not rheumatoid arthritis (RA) or ankylosing spondylitis (AS).
- After accounting for adult body size, the genetically predicted direct effect of childhood body size remained strong for PsA and SLE, whereas evidence of an effect on gout weakened.

**HOW THIS STUDY MIGHT AFFECT RESEARCH, PRACTICE OR POLICY**

- Results of this study suggest that the risk conferred from having a larger body size during childhood may not be fully reversible even when a healthy size is achieved in adulthood.
- How the immune system is altered by early life adiposity to increase risk of PsA and SLE is unclear. Differential effect estimates of body size on each inflammatory arthritis (PsA vs AS, SLE vs RA) may help shed light on their unique pathophysiology in future studies.

## Introduction

Obesity is strongly associated with inflammation and inflammatory arthritis. In rheumatoid arthritis (RA), excess adiposity is associated with increased risk of disease, more severe synovitis, greater clinical disease activity and reduced therapeutic response.^1–3^ Obesity has additionally been linked with increased risk of psoriatic disease,^4 5^ gout^6^ and systemic lupus erythematosus (SLE).^7^ Although pathophysiological mechanisms differ across these conditions, excess inflammation is common to all and the proinflammatory effects of excess adiposity may influence shared immune pathways directly^8^ or indirectly.^9^ Furthermore, this important risk factor is exacerbated by the increasing prevalence of childhood obesity, which is a growing and global public health crisis.^10^

Most obesity research in inflammatory arthritis have been limited by investigations into cross-sectional measures of adult body mass index (BMI) (eg, at diagnosis or treatment initiation) despite the potential for body size to change over the life course. For instance, individuals who are lean in childhood may become overweight in adulthood or vice versa. Untangling the respective effects of early life and adult adiposity on disease risk can inform disease pathophysiology but also interventions. If the effect of early life body size on a given disease is entirely attributed to a persistent effect of individuals remaining overweight across the life course, then any adverse consequences of childhood obesity may be mitigated (or even potentially reversed) by attaining a healthy weight in adolescence and adulthood. Alternatively, if childhood obesity has an immutable effect on disease risk, then intervention may be more effectively targeted during early life rather than adulthood. The importance of age-specific intervention has been proposed in the literature, for example, with suggestive evidence that weight loss in adulthood through bariatric surgery reducing risk of gout^11^ and psoriasis but not psoriatic arthritis (PsA)^12^ or RA.^13^

Studying the effects of adiposity over the life course has challenges even when repeated measurements are available. Traditional observational designs can be limited by reverse causation (ie, obesity can be cause and consequence of disease) and confounding (eg, from lifestyle factors such as cigarette smoking). Mendelian randomisation (MR) can help with these challenges by using genetic variants as instrumental variables to estimate the effect of an exposure on an outcome. Since variants are randomly allocated at conception, MR is less susceptible to reverse causation and confounding.^14 15^

We applied two-sample MR in this study to examine the effects of genetically predicted childhood and adult body size on inflammatory arthritis, namely RA, ankylosing spondylitis (AS), PsA, gout and SLE. We next used multivariable MR to assess whether genetically predicted childhood body size influenced disease risk after accounting for adult body size. This allowed us to separate whether childhood body size has a direct effect on disease risk, or if it may be attributed to an indirect effect through adult body size.

## Methods

### Genetic instruments for childhood and adult body size

Single-nucleotide polymorphisms (SNPs) associated with childhood and adult body size were selected from a genome-wide association study (GWAS) undertaken in the UK Biobank^16^ as described in a previous study.^17^ In brief, GWAS was limited to 453 169 individuals of European descent with both early and adult body size assessments. Participants recalled their body size at age 10 as ‘thinner’, ‘about average’ or ‘plumper’. Adult body size was determined using BMI at a mean age of 57 years and, to facilitate comparison, split into three groups to match the proportions for childhood body size.

Genetic instruments for these body size definitions were previously validated against BMI data from three independent populations: the Avon Longitudinal Study of Parents and Children,^17^ the Young Finns Study^18^ and the Trøndelag Health (HUNT) study.^19^ Furthermore, genetic associations for childhood body size were more strongly correlated with childhood obesity (rG=0.85) than adult BMI (rG=0.64) from large independent studies.^17^ Equally, genetic correlation for adult body size was stronger with adult BMI (rG=0.96) than childhood obesity (rG=0.67).

### GWA studies of inflammatory arthritis

RA genetic associations were obtained from a GWAS meta-analysis including 22 350 cases and 74 823 controls.^20^ All RA cases fulfilled the 1987 American College of Rheumatology (ACR) criteria, or the 2010 ACR/European League Against Rheumatism criteria, or were diagnosed with RA by a rheumatologist; 77% were seropositive for anti-CCP antibodies or rheumatoid factor. The AS GWAS involved 9069 cases fulfilling the modified New York criteria and 13 578 controls.^21^ The PsA GWAS included 3609 cases and 9192 controls; the majority satisfied the ClASsifiaction criteria for Psoriatic ARthritis criteria, but some were recruited prior to its publication.^22^ Genetic associations for gout were taken from a meta-analysis of 13 179 cases and 763 813 controls.^23^ The SLE GWAS comprised 5201 cases (ACR classification criteria) and 9066 controls.^24^ All inflammatory arthritis GWA studies used populations of European descent, except gout where >97% were European.

### Instrument identification and data harmonisation

We selected independent (linkage disequilibrium threshold of r^2^ <0.001 using PLINK and a reference panel of 10 000 randomly selected individuals of European ancestry from UK Biobank) genome-wide significant (p<5×10^−8^) SNPs, resulting in 313 childhood and 580 adult body size instruments. This larger reference panel was used because it allows more accurate calculation of linkage disequilibrium between variants compared with only using European individuals from the 1000 genomes project.^25^ For multivariable MR, we repeated LD clumping for the combined set of SNPs from both exposures. All effect alleles were checked to be on the forward strand; ambiguous palindromes were not excluded. Where possible, SNPs absent in one of the exposure-outcome sets were proxied using variants in linkage disequilibrium (r^2^ >0.8). We calculated F statistics for each exposure in univariable MR, and conditional F statistics in multivariable MR, with F statistics >10 considered suggestive of adequate instrument strength.^26^

### Statistical analysis

**Figure 1:**
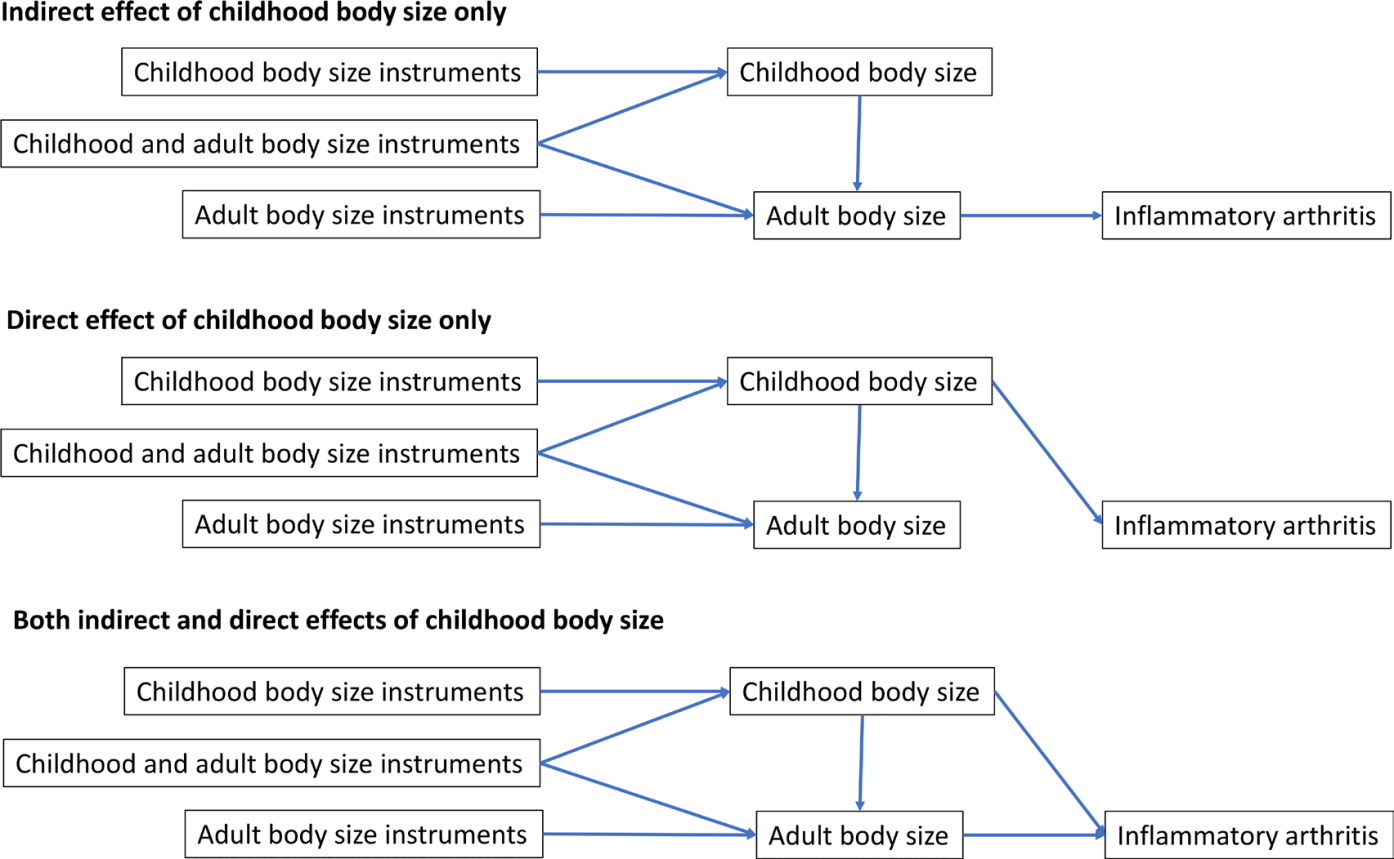
Directed acyclic graphs summarising analyses of childhood body size and inflammatory arthritis outcomes in adulthood. (Top) childhood body size has an indirect effect on disease risk through body size in adulthood, (middle) childhood body size has a direct effect on disease risk independent of adult body size, and (bottom) childhood body size exerts both direct and indirect effects on disease risk in adulthood.

We first applied univariable MR to assess the total effect of genetically predicted childhood and adult body sizes on each disease outcome. The inverse variance weighted (IVW) method was used for the primary analysis, which provides a weighted average of variant effects analogous to meta-analysis.^27^ We then used multivariable MR to estimate the direct effect of childhood body size by accounting for adult body size as an additional exposure in our model.^28^ Directed acyclic graphs summarising these analyses are shown in figure 1.

### Sensitivity analyses

We used the weighted median,^29^ weighed mode^30^ and MR Egger^31^ methods to test the robustness of univariable IVW estimates to horizontal pleiotropy—a potential source of bias in MR studies. We used multivariable MR Egger for this purpose in the multivariable setting.

To test for potential misspecification of each exposure, we used Steiger filtering^32^ to exclude childhood body size SNPs that explained greater adult body size variance and vice versa. Univariable MR analyses were then repeated using the selected SNPs. Multivariable analyses require effect estimates for instruments of both exposures thus was not amenable to Steiger filtering (ie, if an instrument is removed by Steiger filtering for one exposure then it is still retained in the multivariable model).

The AS GWAS in the primary analysis had a relatively smaller number of variants; we, therefore, used a GWAS for AS of 1462 cases (defined by International Classification of Diseases () 10 code, M45) and 164 682 controls from FinnGen (release 5). Genetic correlation was high (rG=0.97) with the AS GWAS using modified New York criteria cases.^21^

The gout GWAS comprised a mixed ancestry population (>97% European), which may introduce bias through population structure. We repeated the gout analysis using an earlier GWAS with 2115 cases and 67 259 controls of European ancestry.^33^

### Supplementary analyses

We also performed two supplementary analyses—using psoriasis and serum urate as outcomes—to facilitate interpretation of primary analysis findings. The FinnGen psoriasis GWAS included 4510 cases (ICD10 code L40, rG=1.00 with gold-standard GWAS which had limited number of variants^34^) and 212 242 controls. The urate GWAS was of 110 347 adults with one SD equivalent to 1.33 mg/dL.^33^

Lastly, we used osteoarthritis (OA) as a negative control. We hypothesised that childhood body size influences inflammatory arthritis through immune/inflammatory pathways, therefore, it should not have a direct effect on OA after accounting for adult body size as it is not a primary inflammatory disease. Genetic data were taken from a GWAS meta-analysis comprising 177 517 OA cases defined using ICD codes and 649 173 controls.^35^ All analyses were performed in R using the TwoSampleMR V.0.5.6 and MVMR V.0.3 packages.^28 36^

## Results

### Total effects of childhood and adult body size on inflammatory arthritis

**Figure 2:**
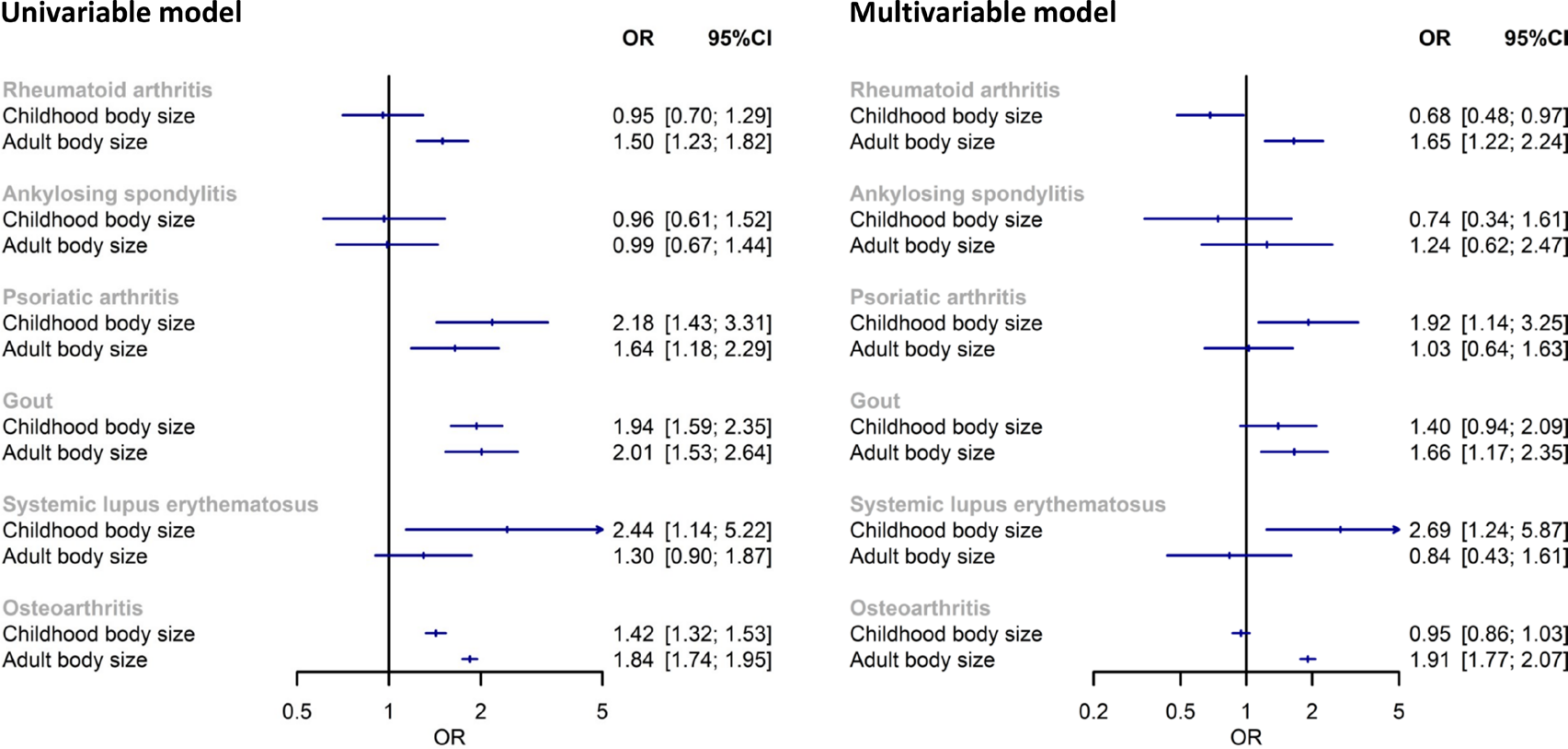
Mendelian randomisation estimates of the effect of childhood and adult body size on inflammatory arthritis and osteoarthritis as the negative control. (Left) Univariable models of each exposure in turn and (right) multivariable models including both.

The F statistic for childhood and adult body size variants were 30 and 23, respectively, suggesting adequate instrument strength. Univariable IVW estimates for the total effect of childhood body size on RA and AS were compatible with no effect (figure 2A). Each increase in childhood body size category increased risk of PsA (OR 2.18; 95% CI 1.43 to 3.31), gout (OR 1.94; 95% CI 1.59 to 2.35) and SLE (OR 2.44; 95% CI 1.14 to 5.22).

Greater adult body size increased risk of RA (OR 1.50; 95% CI 1.23 to 1.82), PsA (OR 1.64; 95% CI 1.18 to 2.29) and gout (OR 2.01; 95% CI 1.53 to 2.64), whereas the CI for SLE included the null (OR 1.30; 95% CI 0.90 to 1.87). Estimate for the effect of adult body size on AS was compatible with no effect (OR 0.99; 95% CI 0.67 to 1.44).

There was statistical evidence of heterogeneity across all analyses, but MR Egger and weighted median/mode estimates were generally consistent with IVW in causal direction with expectedly reduced precision (online supplemental tables S1–S3).

10.1136/rmdopen-2022-002321.supp1 Supplementary data

IVW repeated using Steiger filtered SNPs (236 childhood and 493 adult) did not produce meaningfully different results (online supplemental figure S1).

Analyses repeated using FinnGen GWAS of AS (which had better SNP coverage) and gout (European ancestry) showed similar results as the primary analysis (online supplemental figure S2).

### Direct effects of childhood and adult body size

Conditional F statistics in multivariable MR were 11–12 for childhood and 13–14 for adult body size instruments across all analyses, except for AS (10 and 8, respectively) (online supplemental table S4).

After accounting for adult body size in the multivariable MR model, evidence of an effect of childhood body size remained on risk of PsA (OR 1.92; 95% CI 1.14 to 3.25) and SLE (OR 2.69; 95% CI 1.24 to 5.87), whereas evidence was reduced and included the null for gout (OR 1.40; 95% CI 0.94 to 2.09) (figure 2B).

Multivariable models were performed for RA and AS for completeness; weak evidence of a total effect for childhood body size (univariable model) would not produce a meaningful direct effect after accounting for adult body size.

Multivariable MR Egger estimates for childhood body size were broadly similar to the IVW analysis (online supplemental tables S5 and S6).

### Supplementary analyses

To contextualise results of the above primary analyses, we additionally performed supplementary analyses using serum urate and psoriasis as outcomes.

Each increase in childhood body size was associated with elevated serum urate in univariable MR (beta=0.39 standard deviations (ie, 0.52 mg/dL); 95% CI 0.30 to 0.48) (online supplemental table S1), although the effect was attenuated after adjusting for adult body size in multivariable MR (beta=0.17 (0.23 mg/dL); 95% CI 0.02 to 0.17) (online supplemental tables S4).

Risk of psoriasis increased with childhood (OR 1.39; 95% CI 1.06 to 1.82) and adult body size (OR 2.23; 95% CI 1.78 to 2.80) in univariable MR (online supplemental table S1); childhood estimates included the null in multivariable MR (OR 0.76; 95% CI 0.53 to 1.08) (online supplemental table S4).

Both childhood (OR 1.42; 95% CI 1.32 to 1.53) and adult body size (OR 1.84; 95% CI 1.74 to 1.95) were associated with OA. However, childhood body size did not have an independent effect when adjusting for adult size in the multivariable MR model (OR 0.95; 95% CI 0.86 to 1.03) (figure 2).

## Discussion

In this MR study, we showed that larger childhood body size (as a proxy for adiposity) increased the risk of PsA, gout and SLE. Evidence of these effects persisted even after accounting for adult body size, suggesting that the risk from higher childhood size may not be fully reversable even when a healthy size is achieved in adulthood.

Few studies have examined the role of childhood adiposity on risk of rheumatic diseases and, to our knowledge, none have examined its effect independent of adult body size for outcomes evaluated in this study using human genetics. The independent effect of childhood body size suggests that components of the immune system are irreversibly disrupted to increase PsA and SLE susceptibility. By contrast, an analogous MR study of coronary heart disease and type 2 diabetes showed that the detrimental effects of childhood body size were almost fully mediated through adult body size,^17^ which is compatible with our understanding that their disease mechanisms (atherosclerosis and insulin resistance) are at least partially reversible. Our analysis of the non-inflammatory control, OA, concurs with results of this prior study; one way through which excess adiposity influences OA is through mechanical load that is reversable with weight loss.

Exactly how the immune system is altered by early life adiposity to increase the risk of inflammatory arthritis is not known and made further complex by the distinct pathomechanisms behind PsA and SLE. PsA shares pathophysiology with AS (both in the spondyloarthritis family) and clinical features with RA (peripheral arthritis), yet its risk profile from obesity bears greater resemblance with gout. This highlights the importance of the metabolic syndrome in the PsA disease process and is consistent with the high prevalence of these comorbidities.^37^ Additional exploratory analysis showed that childhood body size had a comparatively smaller effect on psoriasis risk and, in contrast to PsA, had no independent effect after accounting for adult body size. This may suggest childhood body size to be a unique causal risk factor for PsA, although a direct matched comparison of PsA and cutaneous-only psoriasis would be required. A prior trial of bariatric surgery in adulthood showed reduced risk of psoriasis but not PsA, which supports our findings.^12^

We did not find evidence to suggest that body size either in childhood or adulthood is a causal risk factor for AS. Although obesity has been observationally associated with poorer treatment response in AS,^38^ no studies have implicated it as an aetiological risk factor. This is interesting since abnormal immune response to mechanostress (which increases with weight) is thought to be shared by members of the spondyloarthritis family.^39^ Obesity and the metabolic syndrome may contribute unique inflammatory stimuli in PsA that warrants further study. More recent studies suggested that obesity may influence T-helper cell differentiation.^8^ Altered immune cell populations and function, and their interactions with other environmental risk factors, may explain differences observed herein. However, mechanistic explanations for our results are unknown and can only be speculated on.

Unlike PsA and AS—the ‘MHC-I-opathies’^39^—SLE and RA are both associated with major histocompatibility complex (MHC) II genes, autoantibodies and a female preponderance. Greater adult body size appears to increase the risk of both RA and SLE—findings that are consistent with existing literature.^2^ Excess adiposity in girls and young women disrupts sex hormones that play important roles in immune (eg, B cell) function.^40 41^ Further studies are required to examine why childhood body size should uniquely increase SLE risk. The seemingly protective direct effect of childhood body size on RA in multivariable MR is likely spurious given the lack of total effect in the univariable model.

Obesity, and upstream causative lifestyle factors such as diet, are recognised to influence the gut microbiome,^42 43^ which has in turn been implicated in the pathogenesis of rheumatic diseases.^44^ Prior studies have shown that the gut microbiota reaches an adult-like configuration in early childhood,^45^ thus, early life obesity may influence the adult immune system through the microbiome. Immune mechanisms are likely involved since childhood body size had no direct effect on OA risk.

Hyperuricaemia is the leading risk factor for gout that is at least partially reversible with lifestyle changes or urate lowering drugs. This is consistent with multivariable MR results, where the effect of childhood body size on gout and urate were attenuated after accounting for adult size. Although CIs from the IVW multivariable MR analysis of gout included the null, our results cannot rule out a small direct effect of childhood adiposity.

A key strength of the two-sample MR design is that it leverages large sample sizes of existing GWA studies, while many rarer rheumatic diseases would be challenging to study in cohort designs. Assuming instrumental variable assumptions are met, MR estimates the causal exposure–outcome relationship with less bias from confounding or reverse causation. There were, however, limitations. A main source of potential bias in MR studies is horizontal pleiotropy; we examined this using a host of MR methods that provided consistent results to the main analysis. Body sizes were solely derived from the UK Biobank, which is recognised to have highly selected participants. Replication in other populations, particularly different ethnic ancestries, is required. Childhood body size was by recall which may be prone to bias. However, prior validation studies in separate populations support the ability of these instruments to separate childhood and adult adiposity^17–19^; we additionally filtered variants to reduce exposure misclassification. An additional strength of MVMR is that it allows the effect of multiple exposures to be simultaneously estimated in the presence of bidirectional relationships.^46^ Lastly, inferences drawn from our results apply to disease onset and may not extrapolate to prognosis.

In conclusion, we showed that larger childhood body size increases the risk of PsA and SLE independently of adult body size. This increased risk may not be fully reversible even when a healthy body size is achieved in adulthood. Preventing and managing childhood obesity may be a good strategy to reduce disease risk in susceptible groups. Further studies of the immune mechanism through which childhood adiposity independently influences adult disease risk is needed. Differing effects of body size on each inflammatory arthritis (eg, PsA vs AS, SLE vs RA) may also shed light on their unique pathophysiology in future studies.

## Data Availability

All data relevant to the study are included in the article or uploaded as online supplemental information. All data used in this study are publicly available, with relevant citations detailed. Genetic instruments for all analyses are available at https://doi.org/10.48420/19940642.
