# Supplementary material for: Separating the effects of childhood and adult body size on inflammatory arthritis: a Mendelian randomisation study
Source: RMD Open. 2022 Aug 22;8(2):e002321. doi: 10.1136/rmdopen-2022-002321 (PMC9403135; doi:10.1136/rmdopen-2022-002321)
Supplement: Supplementary data [file rmdopen-2022-002321supp001.pdf]

Supplementary materials for

Separating the effects of childhood and adult body size on inflammatory arthritis: a Mendelian randomisation study

Sizheng Steven Zhao[1,2], John Bowes[3], Anne Barton[3], George Davey Smith[2,4], Tom G Richardson[2,4,5]

1 Centre for Epidemiology Versus Arthritis, Division of Musculoskeletal and Dermatological Sciences, School of Biological Sciences, Faculty of Biology Medicine and Health, The University of Manchester, Manchester Academic Health Science Centre, Manchester, UK;

2 Medical Research Council Integrative Epidemiology Unit at the University of Bristol, University of Bristol, UK;

3 Centre for Genetics and Genomics Versus Arthritis, Division of Musculoskeletal and Dermatological Sciences, School of Biological Sciences, Faculty of Biology Medicine and Health, The University of Manchester, Manchester Academic Health Science Centre, Manchester, UK;

4 Population Health Sciences, Bristol Medical School, University of Bristol, Oakfield House, Oakfield Grove, Bristol, UK;

5 Novo Nordisk Research Centre, Headington, Oxford, UK.

Zhao SS, *et al. RMD Open* 2022; 8:e002321. doi: 10.1136/rmdopen-2022-002321

Contents

Table S1: Heterogeneity tests of univariable MR using the inverse variance weighted method.......... 3
Table S2: Univariable MR sensitivity analyses using pleiotropy robust methods. 4
Table S3: Intercepts for univariable MR Egger. 7
Figure S1: Univariable MR primary analysis using Steiger filtered SNPs to reduce exposure misclassification. 8
Figure S2: Sensitivity analyses using FinnGen data with improved variant-coverage. 9
Table S4: Conditional F statistics in multivariable MR 9
Table S5: Multivariable MR using inverse variance weighted and Egger methods. 10
Table S6: Multivariable MR Egger intercepts.. 11
References 12

Table S1: Heterogeneity tests of univariable MR using the inverse variance weighted method.

Outcome	Exposure	Q	p-value
AS (FinnGen)	Adult	642	1.66E-08
AS (FinnGen)	Childhood	318	1.09E-03
AS (IGAS)	Adult	140	2.48E-18
AS (IGAS)	Childhood	218	1.03E-34
Gout (FinnGen)	Adult	691	5.75E-12
Gout (FinnGen)	Childhood	322	6.55E-04
Gout (Tin)	Adult	2162	5.10E-226
Gout (Tin)	Childhood	438	7.55E-14
Psoriatic arthritis	Adult	512	2.11E-06
Psoriatic arthritis	Childhood	331	9.35E-08
Rheumatoid arthritis	Adult	1177	2.63E-52
Rheumatoid arthritis	Childhood	1085	4.60E-95
SLE	Adult	879	1.71E-30
SLE	Childhood	1318	1.36E-148
Urate	Adult	1150	4.50E-88
Urate	Childhood	348	2.24E-11
Psoriasis (FinnGen)	Adult	546	2.15E-03
Psoriasis (FinnGen)	Childhood	285	4.21E-02
Osteoarthritis	Adult	854	4.67E-24
Osteoarthritis	Childhood	558	4.61E-26
AS, ankylosing spondylitis; IGAS, International Genetics of Ankylosing Spondylitis Consortium; SLE, systemic lupus erythematosus.			

Zhao SS, *et al. RMD Open* 2022; 8:e002321. doi: 10.1136/rmdopen-2022-002321

Table S2: Univariable MR sensitivity analyses using pleiotropy robust methods.

Outcome	Exposure	Method	No of SNPs	OR*	Lower 95%CI	Upper 95%CI	P-value
AS (FinnGen)	Adult	IVW	456	1.292	0.856	1.948	0.222
AS (FinnGen)	Adult	MR Egger	456	1.301	0.417	4.060	0.651
AS (FinnGen)	Adult	Weighted median	456	1.444	0.741	2.811	0.280
AS (FinnGen)	Adult	Weighted mode	456	3.196	1.027	9.946	0.045
AS (FinnGen)	Childhood	IVW	246	0.728	0.453	1.170	0.190
AS (FinnGen)	Childhood	MR Egger	246	1.146	0.405	3.239	0.798
AS (FinnGen)	Childhood	Weighted median	246	1.400	0.666	2.944	0.375
AS (FinnGen)	Childhood	Weighted mode	246	1.333	0.468	3.792	0.591
AS (IGAS)	Adult	IVW	25	0.987	0.674	1.445	0.946
AS (IGAS)	Adult	MR Egger	25	1.696	0.534	5.380	0.379
AS (IGAS)	Adult	Weighted median	25	1.464	1.115	1.923	0.006
AS (IGAS)	Adult	Weighted mode	25	1.557	1.044	2.322	0.040
AS (IGAS)	Childhood	IVW	22	0.963	0.608	1.524	0.872
AS (IGAS)	Childhood	MR Egger	22	0.706	0.255	1.959	0.512
AS (IGAS)	Childhood	Weighted median	22	1.022	0.824	1.269	0.842
AS (IGAS)	Childhood	Weighted mode	22	1.030	0.852	1.246	0.763
Gout (FinnGen)	Adult	IVW	456	1.835	1.379	2.441	3.07E-05
Gout (FinnGen)	Adult	MR Egger	456	2.175	0.986	4.797	0.055
Gout (FinnGen)	Adult	Weighted median	456	1.739	1.101	2.748	0.018
Gout (FinnGen)	Adult	Weighted mode	456	1.947	0.853	4.444	0.114
Gout (FinnGen)	Childhood	IVW	246	1.624	1.180	2.237	0.003
Gout (FinnGen)	Childhood	MR Egger	246	1.522	0.754	3.072	0.243
Gout (FinnGen)	Childhood	Weighted median	246	1.650	0.969	2.808	0.065
Gout (FinnGen)	Childhood	Weighted mode	246	1.806	0.976	3.344	0.061
Gout (Tin)	Adult	IVW	437	2.011	1.531	2.642	5.26E-07
Gout (Tin)	Adult	MR Egger	437	2.193	1.001	4.800	0.050

Gout (Tin)	Adult	Weighted median	437	2.301	1.775	2.983	3.15E-10
Gout (Tin)	Adult	Weighted mode	437	2.348	1.427	3.863	0.001
Gout (Tin)	Childhood	IVW	240	1.935	1.594	2.349	2.44E-11
Gout (Tin)	Childhood	MR Egger	240	2.518	1.667	3.802	1.71E-05
Gout (Tin)	Childhood	Weighted median	240	2.184	1.654	2.885	3.71E-08
Gout (Tin)	Childhood	Weighted mode	240	2.241	1.493	3.364	1.28E-04
Psoriatic arthritis	Adult	IVW	374	1.645	1.182	2.289	0.003
Psoriatic arthritis	Adult	MR Egger	374	2.053	0.786	5.361	0.143
Psoriatic arthritis	Adult	Weighted median	374	1.730	1.038	2.884	0.036
Psoriatic arthritis	Adult	Weighted mode	374	1.556	0.671	3.605	0.303
Psoriatic arthritis	Childhood	IVW	208	2.177	1.432	3.311	2.75E-04
Psoriatic arthritis	Childhood	MR Egger	208	2.633	1.051	6.598	0.040
Psoriatic arthritis	Childhood	Weighted median	208	1.730	0.928	3.228	0.085
Psoriatic arthritis	Childhood	Weighted mode	208	1.740	0.815	3.715	0.154
Rheumatoid arthritis	Adult	IVW	524	1.497	1.234	1.816	4.22E-05
Rheumatoid arthritis	Adult	MR Egger	524	1.241	0.729	2.112	0.426
Rheumatoid arthritis	Adult	Weighted median	524	1.184	0.891	1.572	0.244
Rheumatoid arthritis	Adult	Weighted mode	524	1.010	0.666	1.532	0.962
Rheumatoid arthritis	Childhood	IVW	281	0.955	0.705	1.294	0.766
Rheumatoid arthritis	Childhood	MR Egger	281	1.083	0.558	2.100	0.814
Rheumatoid arthritis	Childhood	Weighted median	281	0.958	0.681	1.349	0.807
Rheumatoid arthritis	Childhood	Weighted mode	281	0.829	0.533	1.289	0.405
SLE	Adult	IVW	448	1.300	0.901	1.878	0.161
SLE	Adult	MR Egger	448	0.642	0.215	1.910	0.426
SLE	Adult	Weighted median	448	1.512	0.928	2.462	0.097
SLE	Adult	Weighted mode	448	0.994	0.441	2.241	0.989
SLE	Childhood	IVW	240	2.433	1.134	5.219	0.022
SLE	Childhood	MR Egger	240	4.849	0.887	26.503	0.070
SLE	Childhood	Weighted median	240	1.518	0.817	2.819	0.187
SLE	Childhood	Weighted mode	240	1.296	0.627	2.681	0.485

Urate	Adult	IVW	343	0.429	0.324	0.534	8.84E-16
Urate	Adult	MR Egger	343	0.457	0.152	0.761	0.004
Urate	Adult	Weighted median	343	0.450	0.328	0.571	4.20E-13
Urate	Adult	Weighted mode	343	0.366	0.180	0.553	1.43E-04
Urate	Childhood	IVW	191	0.390	0.299	0.482	5.07E-17
Urate	Childhood	MR Egger	191	0.505	0.304	0.706	1.76E-06
Urate	Childhood	Weighted median	191	0.425	0.302	0.548	1.37E-11
Urate	Childhood	Weighted mode	191	0.336	0.160	0.511	2.32E-04
Psoriasis (FinnGen)	Adult	IVW	456	2.232	1.777	2.803	4.96E-12
Psoriasis (FinnGen)	Adult	MR Egger	456	2.264	1.204	4.257	0.012
Psoriasis (FinnGen)	Adult	Weighted median	456	2.211	1.566	3.120	6.38E-06
Psoriasis (FinnGen)	Adult	Weighted mode	456	2.106	1.043	4.253	0.038
Psoriasis (FinnGen)	Childhood	IVW	246	1.388	1.060	1.818	0.017
Psoriasis (FinnGen)	Childhood	MR Egger	246	1.180	0.653	2.132	0.584
Psoriasis (FinnGen)	Childhood	Weighted median	246	1.145	0.716	1.832	0.572
Psoriasis (FinnGen)	Childhood	Weighted mode	246	1.091	0.585	2.034	0.785
Osteoarthritis	Adult	IVW	476	1.840	1.739	1.946	1.06E-100
Osteoarthritis	Adult	MR Egger	476	1.647	1.401	1.937	2.98E-09
Osteoarthritis	Adult	Weighted median	476	1.728	1.580	1.891	9.94E-33
Osteoarthritis	Adult	Weighted mode	476	1.664	1.429	1.937	1.37E-10
Osteoarthritis	Childhood	IVW	248	1.424	1.322	1.535	1.78E-20
Osteoarthritis	Childhood	MR Egger	248	1.470	1.252	1.726	4.28E-06
Osteoarthritis	Childhood	Weighted median	248	1.479	1.348	1.622	1.33E-16
Osteoarthritis	Childhood	Weighted mode	248	1.541	1.334	1.780	1.35E-08

*beta for urate, expressed as standard deviations representing 1.33 mg/dL.
AS, ankylosing spondylitis; IGAS, International Genetics of Ankylosing Spondylitis Consortium; IVW, inverse variance weighted method; OR, odds ratio; SLE, systemic lupus erythematosus.

Table S3: Intercepts for univariable MR Egger.

Outcome	Exposure	MR Egger intercept	P-value
AS (FinnGen)	Adult	0.000	0.989
AS (FinnGen)	Childhood	-0.007	0.338
AS (IGAS)	Adult	-0.007	0.341
AS (IGAS)	Childhood	0.005	0.511
Gout (FinnGen)	Adult	-0.002	0.651
Gout (FinnGen)	Childhood	0.001	0.838
Gout (Tin)	Adult	-0.001	0.817
Gout (Tin)	Childhood	-0.004	0.158
Psoriasis (FinnGen)	Adult	0.000	0.962
Psoriasis (FinnGen)	Childhood	0.002	0.546
Psoriatic arthritis	Adult	-0.003	0.630
Psoriatic arthritis	Childhood	-0.003	0.649
Rheumatoid arthritis	Adult	0.002	0.458
Rheumatoid arthritis	Childhood	-0.002	0.676
Systemic lupus erythematosus	Adult	0.009	0.170
Systemic lupus erythematosus	Childhood	-0.010	0.381
Urate	Adult	0.000	0.860
Urate	Childhood	-0.002	0.231
Osteoarthritis	Adult	0.001	0.261
Osteoarthritis	Childhood	-0.001	0.479

AS, ankylosing spondylitis; IGAS, International Genetics of Ankylosing Spondylitis Consortium.

Figure S1: Univariable MR primary analysis using Steiger filtered SNPs to reduce exposure misclassification.

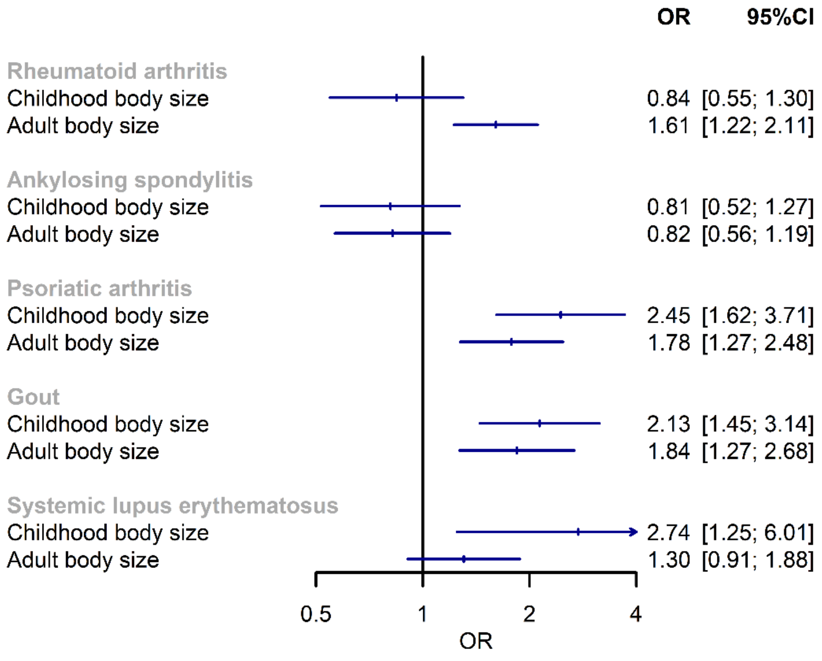

Figure S2: Sensitivity analyses using FinnGen data with improved variant-coverage.

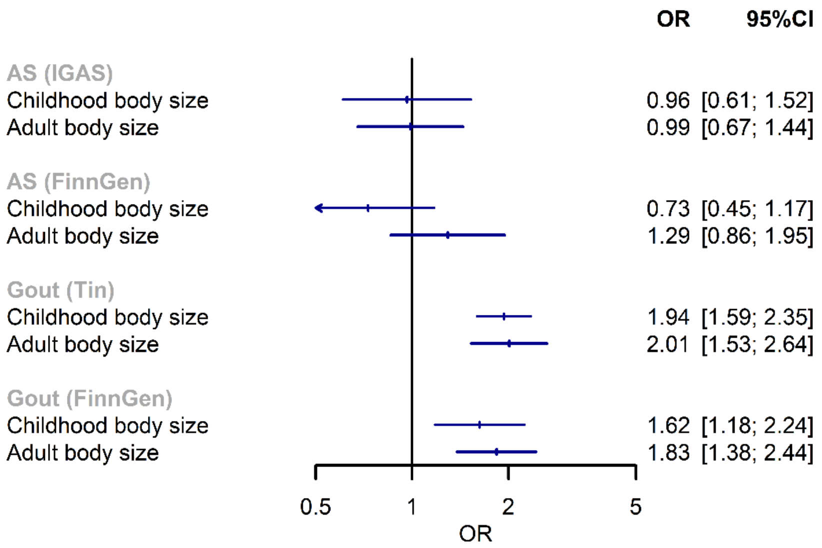

Table S4: Conditional F statistics in multivariable MR

	Childhood body size	Adult body size
Ankylosing spondylitis	9.9	7.6
Gout	11.6	12.9
Psoriasis	11.7	12.8
Psoriatic arthritis	11.8	14.0
Rheumatoid arthritis	11.4	13.0
Systemic lupus erythematosus	11.0	13.1
Urate	11.3	12.8
Osteoarthritis	11.6	13.0

Zhao SS, *et al. RMD Open* 2022; 8:e002321. doi: 10.1136/rmdopen-2022-002321

Table S5: Multivariable MR using inverse variance weighted and Egger methods.

Outcome	Exposure	Method	No of SNPs	OR*	Lower 95%CI	Upper 95%CI	P-value
Ankylosing spondylitis	Adult	IVW	38	1.200	0.631	2.284	0.582
Ankylosing spondylitis	Childhood	IVW	38	0.782	0.383	1.597	0.504
Gout	Adult	IVW	558	1.658	1.167	2.354	0.005
Gout	Childhood	IVW	558	1.399	0.937	2.089	0.101
Psoriasis	Adult	IVW	577	2.432	1.801	3.283	1.08E-08
Psoriasis	Childhood	IVW	577	0.773	0.545	1.096	0.148
Psoriatic arthritis	Adult	IVW	474	1.026	0.645	1.632	0.914
Psoriatic arthritis	Childhood	IVW	474	1.921	1.137	3.245	0.015
Rheumatoid arthritis	Adult	IVW	661	1.650	1.216	2.241	0.001
Rheumatoid arthritis	Childhood	IVW	661	0.683	0.481	0.969	0.033
Systemic lupus erythematosus	Adult	IVW	564	0.837	0.437	1.602	0.591
Systemic lupus erythematosus	Childhood	IVW	564	2.688	1.238	5.838	0.013
Urate	Adult	IVW	443	0.344	0.205	0.483	1.77E-06
Urate	Childhood	IVW	443	0.173	0.015	0.332	0.033
Osteoarthritis	Adult	IVW	597	1.911	1.768	2.066	1.68E-49
Osteoarthritis	Childhood	IVW	597	0.945	0.864	1.035	0.224
Ankylosing spondylitis	Adult	MR Egger	38	1.148	0.601	2.193	0.678
Ankylosing spondylitis	Childhood	MR Egger	38	0.776	0.381	1.581	0.489
Gout	Adult	MR Egger	558	1.660	1.168	2.360	0.005
Gout	Childhood	MR Egger	558	1.395	0.931	2.088	0.107
Psoriasis	Adult	MR Egger	577	2.453	1.816	3.314	8.26E-09
Psoriasis	Childhood	MR Egger	577	0.758	0.534	1.077	0.123
Psoriatic arthritis	Adult	MR Egger	474	1.049	0.659	1.671	0.840
Psoriatic arthritis	Childhood	MR Egger	474	1.851	1.093	3.135	0.022
Rheumatoid arthritis	Adult	MR Egger	661	1.664	1.225	2.259	0.001

Zhao SS, *et al. RMD Open* 2022; 8:e002321. doi: 10.1136/rmdopen-2022-002321

Rheumatoid arthritis	Childhood	MR Egger	661	0.668	0.470	0.950	0.025
Systemic lupus erythematosus	Adult	MR Egger	564	0.862	0.449	1.656	0.656
Systemic lupus erythematosus	Childhood	MR Egger	564	2.553	1.165	5.594	0.020
Urate	Adult	MR Egger	443	0.346	0.206	0.486	1.71E-06
Urate	Childhood	MR Egger	443	0.169	0.009	0.330	0.039
Osteoarthritis	Adult	MR Egger	597	1.919	1.775	2.074	6.13E-50
Osteoarthritis	Childhood	MR Egger	597	0.939	0.857	1.028	0.172

*beta for urate, expressed as standard deviations representing 1.33 mg/dl.
IVW, inverse variance weighted method.

Table S6: Multivariable MR Egger intercepts.

Outcome	Egger intercept	P-value
Ankylosing spondylitis	-0.002	0.287
Gout	-0.0002	0.881
Psoriasis	-0.001	0.314
Psoriatic arthritis	-0.002	0.194
Rheumatoid arthritis	-0.001	0.222
Systemic lupus erythematosus	-0.002	0.383
Urate	-0.0002	0.727
Osteoarthritis	-0.0005	0.101

Zhao SS, *et al. RMD Open* 2022; 8:e002321. doi: 10.1136/rmdopen-2022-002321

References

1. Thomas PE, Aarestrup J, Jacobsen S, Jensen BW, Baker JL. Birthweight, body size, and growth during childhood and risks of rheumatoid arthritis: a large Danish cohort study. Scand J Rheumatol 2021;1–9.

2. Thomas PE, Jensen BW, Sørensen KK, Jacobsen S, Aarestrup J, Baker JL. Early life body size, growth and risks of systemic lupus erythematosus - A large Danish observational cohort study. Semin Arthritis Rheum 2020;50:1507–12.

3. Must A, Jacques PF, Dallal GE, Bajema CJ, Dietz WH. Long-term morbidity and mortality of overweight adolescents. A follow-up of the Harvard Growth Study of 1922 to 1935. N Engl J Med 1992;327:1350–5.

4. Soltani-Arabshahi R, Wong B, Feng B-J, Goldgar DE, Duffin KC, Krueger GG. Obesity in early adulthood as a risk factor for psoriatic arthritis. Arch Dermatol 2010;146:721–6.

Zhao SS, *et al. RMD Open* 2022; 8:e002321. doi: 10.1136/rmdopen-2022-002321
